# Half-life measurements of chemical inducers for recombinant gene expression

**DOI:** 10.1186/1754-1611-8-5

**Published:** 2014-02-01

**Authors:** Nicolo’ Politi, Lorenzo Pasotti, Susanna Zucca, Michela Casanova, Giuseppina Micoli, Maria Gabriella Cusella De Angelis, Paolo Magni

**Affiliations:** 1 Dipartimento di Ingegneria Industriale e dell’Informazione, Università degli Studi di Pavia, via Ferrata 3, Pavia, Italy; 2 Centro di Ingegneria Tissutale, Università degli Studi di Pavia, via Ferrata 3, Pavia, Italy; 3 Centro di Ricerche Ambientali, IRCCS Fondazione Salvatore Maugeri, via Salvatore Maugeri 10, Pavia, Italy

**Keywords:** Degradation rate, Chemical inducers, Synthetic biology, Whole-cell biosensors, BioBrick™, IPTG, ATc, HSL

## Abstract

**Background:**

Inducible promoters are widely spread genetic tools for triggering, tuning and optimizing the expression of recombinant genes in engineered biological systems. Most of them are controlled by the addition of a specific exogenous chemical inducer that indirectly regulates the promoter transcription rate in a concentration-dependent fashion. In order to have a robust and predictable degree of control on promoter activity, the degradation rate of such chemicals should be considered in many applications like recombinant protein production.

**Results:**

In this work, we use whole-cell biosensors to assess the half-life of three commonly used chemical inducers for recombinant *Escherichia coli*: Isopropyl β-D-1-thiogalactopyranoside (IPTG), anhydrotetracycline (ATc) and N-(3-oxohexanoyl)-L-homoserine lactone (HSL). A factorial study was conducted to investigate the conditions that significantly contribute to the decay rate of these inducers. Temperature has been found to be the major factor affecting ATc, while medium and pH have been found to highly affect HSL. Finally, no significant degradation was observed for IPTG among the tested conditions.

**Conclusions:**

We have quantified the decay rate of IPTG, ATc and HSL in many conditions, some of which were not previously tested in the literature, and the main effects affecting their degradation were identified via a statistics-based framework. Whole-cell biosensors were successfully used to conduct this study, yielding reproducible measurements via simple multiwell-compatible assays. The knowledge of inducer degradation rate in several contexts has to be considered in the rational design of synthetic biological systems for improving the predictability of induction effects, especially for prolonged experiments.

## Background

Isopropyl β-D-1-thiogalactopyranoside (IPTG), anhydrotetracycline (ATc) and N-(3-oxohexanoyl)-L-homoserine lactone (HSL) are popular chemical inducers that can be used to exogenously control the transcriptional activity of wild-type or synthetic LacI-, TetR- and LuxR-regulated promoters, respectively [1,2]. They have been used in a wide number of engineered biological systems to manipulate the production of recombinant proteins in bacteria [3-7], yeasts [8,9] and also in mammalian cells [10]. Synthetic biology advances have provided several examples of finely tuned highly complex gene networks that can be predictably controlled by means of IPTG, ATc and HSL [11,12]. Although such chemicals have been successfully used in biotechnological research, problems could occur when experiments are performed for prolonged times, because inducers may degrade and gene expression could change accordingly. This is a common situation when inducible promoters are used to drive or optimize the production process of industrially-relevant genes in recombinant cultures that are to be grown for long times (e.g., days) before achieving the optimal amount of product (e.g., a protein, a drug or a biofuel). Although the use of chemical inducers in a large-scale industrial context is sometimes undesirable, as they can account for non-negligible costs, inducible promoters can be used to explore the optimal gene dosage in laboratory-scale experiments, before being substituted with constitutive promoters with optimal activity [13] or promoters that can be induced by cheaper signals, such as temperature or pH [14]. In this framework, degradation of inducer molecules must be taken into account to predict the actual promoter transcription during the optimization process in the lab.

In the present work, we face these problems by assessing the degradation rate of IPTG, ATc and HSL in *Escherichia coli* culture or sterile broth in two commonly used growth media, which are popular in synthetic biology studies: L-broth (LB) and M9 supplemented with thiamine and casamino acids with glycerol as carbon source (M9). Acidity- and temperature-dependent effects are also studied.

Whole-cell biosensors were used as measurement tools for this purpose. Their essential design principles are reviewed in [15] and can be easily generalized and adapted for the detection of the chemical inducers of interest in this work. Other more precise and sensitive methods could also be used to detect these inducers, such as mass spectrometry, although more expensive in terms of instrumentation [16,17].

IPTG is known to be a highly stable molecule in solution at room temperature [18] and in bacterial cultures under commonly used conditions for recombinant protein production [19,20]. IPTG degradation in cultures and its uptake by different *E. coli* strains was also studied [19], demonstrating substantial differences between strains with or without lactose permease, which is actively involved in IPTG transportation into the cells. However, no reports exist on IPTG stability as a function of temperature, media, pH or combinations of such factors. To our knowledge, no degradation assay has been performed for ATc in media or cultures and, in other modelling works, its degradation rate was assumed to be equal to the one of tetracycline in water [21]. Degradation of HSL and other N-acyl homoserine lactones has been mainly studied as a function of pH [22,23], temperature [22], acyl chain length [22] and of specific lactonase enzymes produced by other microbes [24-26], but no extensive study has been carried out on its degradation in lactonase-free cultures in different *E. coli* growth media. Enzyme-independent lactonolysis was found to be caused by ring opening of the molecule in a pH-dependent manner, with an increasing rate for longer acyl chains and at higher temperature [22]. Such process was found to be reversible only by acidifying the solution at pH < 3.0, thus making this process non-reversible under the standard growth conditions of *E. coli*[22].

Since the advances of synthetic biology are enabling the construction of increasingly complex controllable systems [12] and industrially relevant solutions [27], predictability has become a crucial point for the realization and debugging of customized technologies. With this work, we intend to provide useful data to support the predictable tuning of gene expression in recombinant *E. coli* cultures.

## Methods

### Reagents and media

IPTG (I1284, Sigma Aldrich) and ATc (631310, Clontech) were purchased as 200 mM and 2 mg/ml liquid stocks, respectively, and were routinely stored at −20°C. HSL (K3007, Sigma Aldrich) powder was dissolved in deionized water and filter-sterilized (0.2 μm) to prepare a 2 mM stock solution, routinely stored at −20°C.

LB composition is: 10 g/l NaCl, 5 g/l yeast extract and 10 g/l tryptone. M9 composition is: 11.28 g/l M9 salts (M6030, Sigma), 2 mM MgSO_4_, 0.1 mM CaCl_2_, 2 g/l casamino acids, 1 mM thiamine hydrochloride and 4 ml/l glycerol. They were prepared as described in [28]. When appropriate, the pH was adjusted by adding hydrochloric acid or sodium hydroxide.

### Biosensors

**Figure 1:**
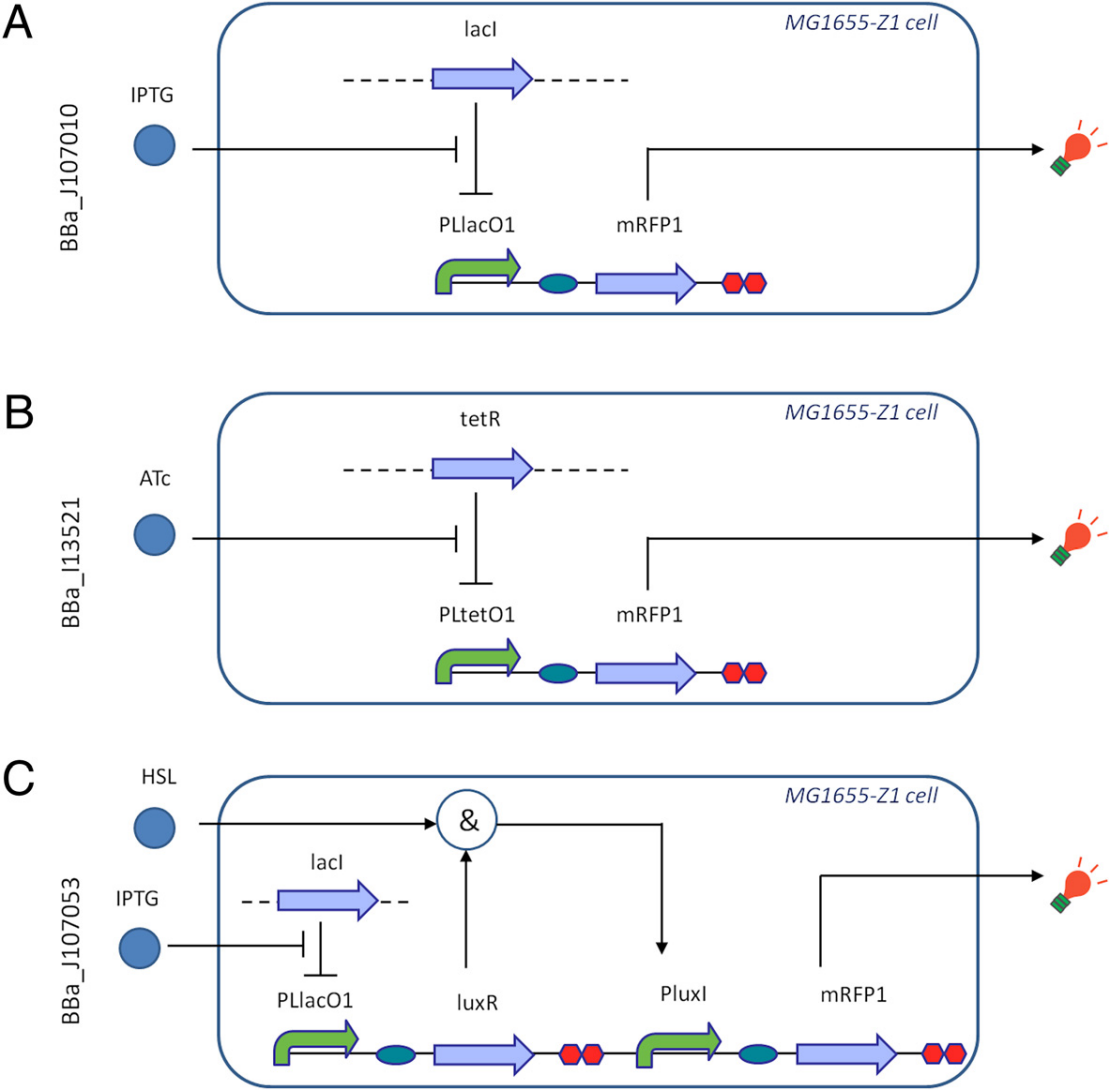
**Biosensors used in this study.** IPTG- **(A)**, ATc- **(B)** and HSL- **(C)** biosensor constructs and functioning. The BioBrick™ code (BBa_code) of the biosensor-encoding sequences and the microbial host are reported for each construct. All of them were used in the pSB3K3 medium-copy BioBrick™ vector backbone with the p15A replication origin and kanamycin resistance marker. Symbols: curved arrows represent promoters, straight arrows represent coding sequences, ovals represent RBSs, octagons represent transcriptional terminators, “&” means that both inducer and gene product are required to activate the promoter, circles represent chemical inducers, bulbs represent a red fluorescence output and finally dashed lines represent MG1655-Z1 genomic DNA. PLlacO1 and PLtetO1 are synthetic promoters that can be repressed by LacI and TetR, respectively. PluxI is the wild type luxI promoter from *Vibrio fischeri* that can be activated by the LuxR-HSL complex. In the HSL-biosensor **(panel C)**, luxR expression was always induced by 500 μM of IPTG, as gene expression is driven by the PLlacO1 promoter.

Figure 1 describes the recombinant *E. coli* bearing the biosensors. The IPTG-, ATc- and HSL-biosensor genetic devices, called BBa_J107010, BBa_I13521 and BBa_J107053, respectively, are BioBrick™ parts from the MIT Registry of Standard Biological Parts (Registry) [29].

They have been assembled into the pSB3K3 medium-copy plasmid (with kanamycin-resistance marker and p15A replication origin) by using the BioBrick™ Standard Assembly procedure [30] and conventional molecular biology techniques [28]. Detailed description about plasmid construction, as well as full DNA sequences, can be found in the individual parts web pages of the Registry [29]. TOP10 (Invitrogen) *E. coli* strain was used as a host for cloning. MG1655-Z1 *E. coli* strain [31] was used as the final host for the biosensing plasmids, as its genome encodes constitutive LacI and TetR over-expression cassettes. Chemically competent TOP10 were transformed according to manufacturer’s instructions, while MG1655-Z1 competent cells were prepared as described in [28] and were heat-shock transformed at 42°C. The described whole-cell biosensors were routinely stored at −80°C in 20% glycerol stocks and they were always grown in selective media with 20 mg/l of kanamycin.

### Sampling

1 mM of IPTG, 50 ng/ml of ATc or 50 nM of HSL were added to 3 ml of sterile medium (*sterile broth*) or to 3 ml of an MG1655-Z1 *E. coli* culture harbouring no plasmids (*cultured broth*), prepared by inoculating 3 ml of sterile medium (LB or M9) with a bacterial colony isolated from a streaked LB agar plate. Sterile and cultured broths, in 15-ml tubes, were incubated with shaking at 220 rpm at 30°C or 37°C. The pH of media was set at 6.0 or 7.0. As a result, each inducer was tested in 16 conditions (cultured/sterile broth; pH 6.0/7.0; 30/37°C; LB/M9 medium). At appropriate time points, 100 μl were taken from the cultured broth and centrifuged (13,000 rpm, 1 min), bacteria-free supernatant was taken and stored at −20°C before analysis. For sterile broth, 100 μl were taken and directly stored at −20°C. For each investigated condition, at least two independent tubes were prepared and sampled in different days. When required, cultured broths were prepared by inoculating a colony of the lacY-deficient strain DB3.1 [32,33] or MC1061 [34] instead of MG1655-Z1.

The growth of cultures was measured by optical density at 600 nm (OD600) in a 96-well microplate (Greiner) in the Infinite F200 reader (Tecan). The presented OD600 measurements are background (sterile media)-subtracted absorbance values, relative to the pathlength of 200 μl of liquid in microplate.

### Biosensor-based fluorescence assays

0.5 ml of selective M9 were inoculated with 1 μl of the appropriate biosensor glycerol stock and incubated for 16 h at 37°C, 220 rpm. Bacteria were 50-fold diluted in fresh selective M9 and grown under the same conditions as before. If using the HSL-biosensor, IPTG (500 μM) was added after 1 h to induce LuxR expression. After 2–3 h from dilution, 190-μl aliquots were transferred into a 96-well microplate (Greiner). Cells at this point are at an OD600 of about 0.03 (exponential growth phase). Wells were induced with 10 μl of properly diluted samples. Standard calibration curves were obtained by inducing the wells with 10 μl of properly diluted inducer amounts. The microplate was incubated in the Infinite F200 reader and it was assayed with the following kinetic cycle, programmed via the i-control™ software (Tecan): linear shaking (3-mm amplitude, 15 s), wait (5 s), OD600 measurement, fluorescence measurement (excitation at 535 nm, emission at 620 nm, gain = 50, to detect RFP signal), repeat cycle every 5 min.

Each calibration curve dilution sample was prepared in the same growth medium of the sampled cultured/sterile broth. A non-fluorescent culture and sterile medium were always included in each experiment to estimate the background fluorescence and absorbance. Calibration curve points were assayed in duplicate, while a single well was used for the unknown samples points (technical replicates of the same sample give highly reproducible measurements with an average CV lower than 7%, data not shown).

When measured values were under the detection limit of the standard curve, experiments were repeated by adding 20 μl of sample to 180 μl of biosensor. When measured values were still under the detection limit of the standard curve (with this dilution, usually 100 μM for IPTG, 10 ng/ml for ATc and 2 nM for HSL), they were set at the detection limit value itself. These detection limits correspond to 10%, 20% and 4% of the IPTG, ATc and HSL initial concentrations.

### Data analysis

The acquired time series were processed as described previously [35,36] to obtain a value proportional to the average RFP protein synthesis rate per cell (S_cell_), which is related to the inducer concentration in the wells. Briefly, raw absorbance and fluorescence time series were background-subtracted over time by using sterile medium and non-fluorescent culture as background to obtain the actual fluorescence (F) and absorbance (OD600) signals in the well, proportional to the per-well RFP molecules and number of bacteria, respectively. The (dF/dt)/OD600 signal was averaged at its steady-state to obtain S_cell_. The steady-state is reached after a time that depends on the dynamics of each specific biosensor. S_cell_ is measured as arbitrary units (AU) of RFP per minute per cell.

The obtained standard calibration curve was fitted with the Hill function S_cell_(I) = δ + α/(1 + (K_m_/I)^n^), where δ + α is the S_cell_ at maximum induction, K_m_ is the inducer concentration that yields S_cell_ = δ + α/2, n is the Hill coefficient, δ is the basic activity of the biosensor in absence of inducer and I is the inducer concentration. The inducer concentration of the unknown samples was computed from their S_cell_ value via the identified Hill function representing the calibration curve. When required, the resulting value was multiplied by the applied dilution factor of the sample.

Each inducer decay curve was expressed as percentage of its value at t = 0 and, for each tested condition, the independently measured time series were pooled in a single time series c(t).

Assuming a first-order exponential decay for c(t), its natural logarithm was fitted with the linear function ln(c(t)) = ln(c(0)) − k * t, where k is the degradation rate of the inducer. Fitting was performed with MATLAB R2011b (MathWorks, Natick, MA) via the *regress* routine, which also computes the 95% confidence intervals of k. When required, inducer half-life was computed as ln(2)/k. The measurement unit of k is h^-1^.

For each inducer, the following linear model with interactions was used to determine the experimental factors that significantly affect the decay rate:

(1) $$\begin{array}{rl} \ln\left(c\left(t\right)\right)=\alpha + & \alpha_{\mathrm{Temp}}\cdot \mathrm{Temp}+\alpha_{\mathrm{pH}}\cdot \mathrm{pH}+\alpha_{\mathrm{Med}}\cdot \mathrm{Med}+\alpha_{\mathrm{Ster}}\cdot \mathrm{Ster}\\ + & \alpha_{\mathrm{Temp}\cdot \mathrm{pH}}\cdot \mathrm{Temp}\cdot \mathrm{pH}+\alpha_{\mathrm{Temp}\cdot \mathrm{Med}}\cdot \mathrm{Temp}\cdot \mathrm{Med}\\ + & \alpha_{\mathrm{Temp}\cdot \mathrm{Ster}}\cdot \mathrm{Temp}\cdot \mathrm{Ster}+\alpha_{\mathrm{pH}\cdot \mathrm{Med}}\cdot \mathrm{pH}\cdot \mathrm{Med}\\ + & \alpha_{\mathrm{pH}\cdot \mathrm{Ster}}\cdot \mathrm{pH}\cdot \mathrm{Ster}+\alpha_{\mathrm{Med}\cdot \mathrm{Ster}}\cdot \mathrm{Med}\cdot \mathrm{Ster}\\ - & (\beta_{t}+\beta_{\mathrm{Temp}}\cdot \mathrm{Temp}+\beta_{\mathrm{pH}}\cdot \mathrm{pH}+\beta_{\mathrm{Med}}\cdot \mathrm{Med}+\beta_{\mathrm{Ster}}\cdot \mathrm{Ster}\\ & \;+\beta_{\mathrm{Temp}\cdot \mathrm{pH}}\cdot \mathrm{Temp}\cdot \mathrm{pH}+\beta_{\mathrm{Temp}\cdot \mathrm{Med}}\cdot \mathrm{Temp}\cdot \mathrm{Med}\\ & \;+\beta_{\mathrm{Temp}\cdot \mathrm{Ster}}\cdot \mathrm{Temp}\cdot \mathrm{Ster}+\beta_{\mathrm{pH}\cdot \mathrm{Med}}\cdot \mathrm{pH}\cdot \mathrm{Med}\\ & \;+\beta_{\mathrm{pH}\cdot \mathrm{Ster}}\cdot \mathrm{pH}\cdot \mathrm{Ster}+\beta_{\mathrm{Med}\cdot \mathrm{Ster}}\cdot \mathrm{Med}\cdot \mathrm{Ster})\cdot t \end{array}$$

This model describes ln(c(t)) behaviour by using time (t) as continuous independent variable and pH, temperature (Temp), sterility (Ster) and medium (Med) as Boolean independent variables assuming the −1 or +1 value, where −1 corresponds to pH 6.0, 30°C, sterile broth and LB, while +1 corresponds to pH 7.0, 37°C, cultured broth and M9, respectively.

The MATLAB *regstats* routine was used to estimate the regression coefficients and their p-value (P). To test if an experimental factor (or interaction between two factors) significantly contributes to inducer degradation, its β coefficient was considered, i.e., the coefficient of the factor*time term. When P < 0.05 for a given coefficient, the related factor (or interaction) is considered to significantly affect the inducer decay rate.

The variability of ln(c(t)) explained by the j-th factor (SS_j_) or an interaction term was quantified and compared with the residual unexplained variability of the following (reference) null model (SS_tot_):

(2) $$\begin{array}{ll} \ln\left(c\left(t\right)\right)=\alpha & +\alpha_{\mathrm{Temp}}\cdot \mathrm{Temp}+\alpha_{\mathrm{pH}}\cdot \mathrm{pH}+\alpha_{\mathrm{Med}}\cdot \mathrm{Med}+\alpha_{\mathrm{Ster}}\cdot \mathrm{Ster}\\ & +\alpha_{\mathrm{Temp}\cdot \mathrm{pH}}\cdot \mathrm{Temp}\cdot \mathrm{pH}+\alpha_{\mathrm{Temp}\cdot \mathrm{Med}}\cdot \mathrm{Temp}\cdot \mathrm{Med}\\ & +\alpha_{\mathrm{Temp}\cdot \mathrm{Ster}}\cdot \mathrm{Temp}\cdot \mathrm{Ster}+\alpha_{\mathrm{pH}\cdot \mathrm{Med}}\cdot \mathrm{pH}\cdot \mathrm{Med}\\ & +\alpha_{\mathrm{pH}\cdot \mathrm{Ster}}\cdot \mathrm{pH}\cdot \mathrm{Ster}+\alpha_{\mathrm{Med}\cdot \mathrm{Ster}}\cdot \mathrm{Med}\cdot \mathrm{Ster}‒\beta_{t}\cdot t \end{array}$$

In particular, SS_tot_, SS_j_ and the residual unexplained variability SSE can be calculated as follows:

(3) $$SS_{\mathrm{tot}}=\sum_{i}{\left(y_{i}-y_{\mathrm{null},i}\right)}^{2}$$

(4) $$SS_{j}=SS_{\mathrm{tot}}‒\sum_{i}{\left(y_{i}‒y_{\mathrm{null},i}+\beta_{j}\cdot X_{\mathrm{ji}}\cdot t_{i}\right)}^{2}$$

(5) $$\mathrm{SSE}=\sum_{i}{\left(y_{i}-y_{\mathrm{full},i}\right)}^{2}$$

where y_i_ are the experimental data, y_full,i_ is the full model (Eq.1) output prediction computed in the i-th experimental point, y_null,i_ is the null model (Eq.2) output prediction computed in the i-th experimental point, β_j_ is the regression coefficient of the j-th factor or interaction term and X_ji_ is the value (−1 or +1) of the j-th factor or interaction term in the i-th experimental point. When X_ji_ is an interaction term, it is computed as the product of the individual factors values (−1 or +1) in the i-th point. As a result, SS_j_ is the variability of the output that is explained by the β_j_⋅X_j_⋅t term alone, thus quantifying the importance of the j-th factor or interaction term. SSE represents the output variability that remains unexplained by the full model (which includes all the considered factors and interaction terms).

When appropriate, the linear model was used without considering cultured broth data and, in this case, sterility was not included as a factor.

### Measurement of ATc with High Performance Liquid Chromatography (HPLC)

HPLC is known to enable ATc measurements via UV detection [37]. Here, a 10 AD/vp HPLC (Shimadzu) was used with a Discovery C18 HPLC column 150 × 4.6 mm, 5 μm (Supelco) and a Diode Array UV Detector SPD-M10AVP (Shimadzu). The column was kept at 25°C. The flow rate of the mobile phase was 0.8 ml/min. Gradient elution was performed with solutions A (0.1% formic acid in water) and B (0.1% formic acid in acetonitrile) following the profile: 0–2 min 10% of B, 2–8 min 10% to 30% of B (linear increase), 8–12 min 30% to 80% of B (linear increase), 12–17 min 80% of B (constant), 17–19 min 80% to 10% of B (linear decrease) and 19–25 min 10% (constant). Each single analysis was run for 25 min with additional 3 min for column conditioning. The injection of 100 μl was performed via automatic injector. A standard calibration curve was prepared in sterile M9 at pH 7.0. The LabSolutions software (Shimadzu) was used to analyze HPLC data. As identified by analyses of standard solutions, ATc retention time is 14.26 min and the maximum absorbance of ATc is observed at a wavelength of 430 nm. Experiments and sampling were performed as described above with the following exceptions: culture broth volume was 7 ml, the initial ATc concentration was 200 ng/ml, at appropriate time points 1.5 ml were taken and filter-sterilized (0.2 μm) for HPLC analysis and 100 μl were taken as described above for analysis via biosensor. All the samples were stored at −20°C before the analyses.

## Results

Additional file 1: Figure S1 shows calibration curves of the IPTG, ATc and HSL biosensors in a representative experiment. Each biosensor yields reproducible measurements of the same samples in different days with a CV of 7%, 21% and 11% for IPTG, ATc and HSL, respectively (see Additional file 1: Figure S2). No relevant difference in biosensor activity can be detected when a known inducer amount was diluted in either exhausted or sterile medium (see Additional file 1: Figure S3), thus demonstrating that these biosensors can be successfully used to detect inducer concentration in sterile or cultured broth conditions.

Table 1 reports the degradation rates of IPTG, ATc and HSL estimated from experimental data in all the investigated conditions, while Additional file 1: Figure S4-S6 show all the data and fitted curves.

**Table 1:** **Degradation rate measurements, expressed as h**^**-1**^**, for IPTG, ATc and HSL in all the tested conditions**

**pH**	**Temperature**	**Sterility**	**Medium**	**k (L; U), IPTG**	**k (L; U), ATc**	**k (L; U), HSL**
6	30°C	Sterile	LB	0.001	0.021	0.001
				(−0.002; 0.005)	(0.018; 0.024)	(−0.001; 0.003)
6	30°C	Sterile	M9	−0.005	0.029	0.021
				(−0.017; 0.007)	(0.022; 0.035)	(0.012; 0.029)
6	37°C	Sterile	LB	0.001	0.039	0.01
				(−0.002; 0.004)	(0.03; 0.049)	(0.007; 0.014)
6	37°C	Sterile	M9	−0.002	0.044	0.034
				(−0.01; 0.006)	(0.042; 0.047)	(0.024; 0.044)
7	30°C	Sterile	LB	0.005	0.017	0.019
				(0; 0.01)	(0.016; 0.019)	(0.013; 0.024)
7	30°C	Sterile	M9	−0.001	0.021	0.085
				(−0.005; 0.004)	(0.012; 0.031)	(0.069; 0.102)
7	37°C	Sterile	LB	−0.004	0.029	0.039
				(−0.011; 0.002)	(0.019; 0.038)	(0.037; 0.042)
7	37°C	Sterile	M9	−0.003	0.041	0.076
				(−0.01; 0.003)	(0.036; 0.046)	(0.037; 0.114)
6	30°C	Cultured	LB	0.116	0.021	−0.006
				(0.09; 0.143)	(0.007; 0.035)	(−0.009; -0.003)
6	30°C	Cultured	M9	0.037	0.026	0.007
				(0.027; 0.048)	(0.018; 0.033)	(0.002; 0.012)
6	37°C	Cultured	LB	0.115	0.051	0.027
				(0.089; 0.141)	(0.035; 0.067)	(0.013; 0.04)
6	37°C	Cultured	M9	0.114	0.053	0.013
				(0.07; 0.158)	(0.032; 0.073)	(0.003; 0.024)
7	30°C	Cultured	LB	0.096	0.02	0.063
				(0.072; 0.12)	(0.006; 0.033)	(0.032; 0.094)
7	30°C	Cultured	M9	0.096	0.031	0.062
				(0.069; 0.122)	(0.011; 0.052)	(0.045; 0.079)
7	37°C	Cultured	LB	0.075	0.042	0.091
				(0.024; 0.126)	(0.021; 0.064)	(0.066; 0.116)
7	37°C	Cultured	M9	0.093	0.058	0.088
				(0.071; 0.114)	(0.038; 0.079)	(0.062; 0.114)

Estimated lower (L) and upper (U) 95% confidence intervals of the measured decay rates (k) are also reported. According to the first-order decay model (see Methods), negative values of k are due to experimental noise and indicate a non-significant decaying trend of the inducer. In this table, cultured broth conditions were tested with the MG1655-Z1 strain.

**Figure 2:**
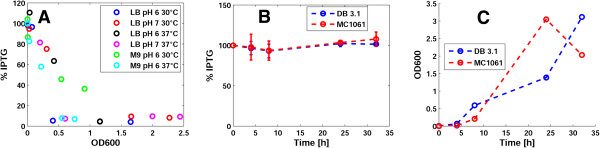
**IPTG measured in the supernatant of cultures of strains with or without lactose permease, as a function of cell density.** Measured IPTG in supernatants of MG1655-Z1 (lacY+) cultured broth conditions as a function of cell density, expressed as OD600 **(A)**. Measured IPTG in supernatants of DB3.1 and MC1061 (lacY-) cultured broth conditions in LB pH 7.0 at 37°C over time **(B)**. Growth of DB3.1 and MC1061, expressed as OD600, in a representative experiment performed in LB pH 7.0 at 37°C **(C)**. In all the panels, circles represent data points, with colours specified in the legends, and error bars represent the 95% confidence intervals of the average.

IPTG is stable over 32 hours in all the sterile broth conditions (the 95% confidence interval of k contains zero in all the conditions, i.e., k is not distinguishable from zero from a statistical point of view), while in all the cultured broth conditions IPTG disappears in the supernatant (see Table 1 and Additional file 1: Figure S4). IPTG uptake by lactose permease (LacY) of the MG1655-Z1 strain could explain the observed phenomenon, as described in [19]. Correlation between OD600 and residual percent IPTG over time (see Figure 2A and Additional file 1: Figure S7) is consistent with this statement.

To further validate this, we measured IPTG in the LB pH 7.0 at 37°C cultured broth condition, inoculated with the DB3.1 or MC1061 strains, both deficient in LacY. Results (see Figure 2B-C) showed that IPTG is not degraded in this condition and the inducer disappearance in the medium of MG1655-Z1 was actually due to uptake by LacY. These results confirm the high stability of the IPTG molecule, previously found in literature in different conditions [16,19,20], and the role of lactose permease in IPTG uptake by LacY + strains. None of the tested environmental factors have been found to affect IPTG stability.

**Figure 3:**
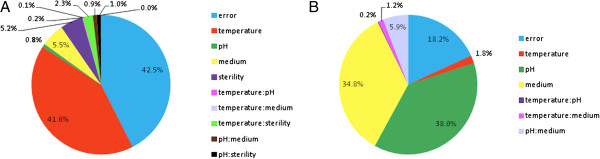
**Quantification of the main factors and their interaction terms contributing to the degradation of ATc (A) and HSL (B).** The pie charts show the variability of the linear model output explained by each of the considered factors and interaction terms (SS_j_, Eq.4). 100% of the pie corresponds to the residual error of the null model (SS_tot_, Eq.3), while the error term represents the residual error that remains unexplained by the full linear model (SSE, Eq.5). Colon indicates an interaction between two factors. Only sterile broth conditions are considered for HSL and, as a result, the sterility factor and its related interaction terms are not present in panel **B**.

ATc is degraded in all the tested conditions with an average half life of about 20 hours (see Table 1). The factors that give a significant contribution in degradation were identified by fitting a linear regression model (see Methods section). According to Table 2, the statistically significant factors (see p-values) are temperature, sterility and medium. Temperature was found to be the main significant factor affecting degradation, while sterility and medium give a minor contribution (see Figure 3A).

**Table 2:** Parameters of the linear model

	** ATc**	
**Parameter**	**Identified value**	**p-value**
α	4.562	1.2e-165
α_Temp_	0.0003582	0.99
α_pH_	0.002346	0.93
α_Med_	0.02576	0.31
α_Ster_	0.01122	0.66
α_Temp·pH_	−0.01358	0.59
α_Temp·Med_	0.02222	0.38
α_Temp·Ster_	0.004408	0.86
α_pH·Med_	0.02403	0.34
α_pH·Ster_	0.01493	0.56
α_Med·Ster_	0.04917	5.4e-2
**β**_**t**_	**0.03398**	**2.7e-52**
**β**_**Temp**_	**0.01076**	**1.5e-12**
β_pH_	−0.001510	0.28
**β**_**Med**_	**0.003901**	**5.4e-3**
**β**_**Ster**_	**0.003825**	**6.4e-3**
β_Temp·pH_	−0.0006518	0.64
β_Temp·Med_	0.0004395	0.75
β_Temp·Ster_	0.002527	7.0e-2
β_pH·Med_	0.001577	0.26
β_pH·Ster_	0.001638	0.24
β_Med·Ster_	0.0002971	0.83
	** HSL**	
**Parameter**	**Identified value**	**p-value**
α	4.441	9.4e-74
α_Temp_	−0.09555	3.5e-2
α_pH_	−0.1136	1.3e-2
α_Med_	−0.1403	2.4e-3
α_Temp·pH_	−0.05275	0.24
α_Temp·Med_	−0.1082	1.8e-2
α_pH·Med_	−0.1057	2.0e-2
**β**_**t**_	**0.03560**	**1.8e-22**
β_Temp_	0.004155	9.1e-2
**β**_**pH**_	**0.01911**	**4.4e-11**
**β**_**Med**_	**0.01827**	**1.8e-10**
β_Temp·pH_	−0.001483	0.54
β_Temp·Med_	−0.003336	0.17
**β**_**pH·Med**_	**0.007504**	**2.9e-3**

Estimated regression coefficients and p-values are reported for the study of ATc and HSL. Only sterile broth conditions were considered for HSL. The regression coefficients β (i.e., the coefficients that are multiplied by time) shown in bold are significantly different from zero (P < 0.05, t-test) and identify the main factors or interaction terms that significantly contribute to inducer decay.

In particular, 42% of the total variability is explained by temperature, while sterility and medium together account for only 11%. The decay rate of ATc is 2-fold higher at 37°C (β + β_Temp_ = 0.045 h^-1^) than at 30°C (β-β_Temp_ =0.023 h^-1^), which corresponds to half-lives of 15 h and 30 h, respectively, without considering the other factors. Conversely, degradation is only 1.26-fold higher in cultured broth or M9 (β + β_Ster_ = 0.038 h^-1^, β + β_Med_ = 0.038 h^-1^) than in sterile broth or LB (β-β_Ster_ = 0.03 h^-1^, β-β_Med_ = 0.03 h^-1^). Conclusions do not substantially change if cultured broth conditions are excluded from the study (see Additional file 1: Table S1 and Figure S8): temperature is confirmed to be the most important factor, while the other statistically significant factors (in this case pH and medium) give a much lower contribution.

HPLC was used to confirm the obtained results of ATc decay in one representative condition (M9 cultured broth at 30°C, pH 7.0), yielding consistent ATc measurements and degradation rates (see Additional file 1: Figure S9).

HSL spans a wide range of decay rates, from zero (i.e., no detectable degradation in 32 hours) to a half-life of about 8 hours (see Table 1). Degradation was firstly studied in sterile broth conditions by fitting a linear regression model, since pH is already known to affect inducer decay and in cultured broth conditions bacteria can change medium acidity during growth (see Additional file 1: Figure S10). Table 2 shows that the identified significant factors affecting HSL degradation are medium and pH with a significant interaction between them. They explain 38% (pH), 35% (Med) and 6% (pH:Med) of the output variability, respectively (see Figure 3B). In particular, HSL degradation is negligible in the LB pH 6.0 condition (β-β_pH_-β_Med_ + β_pH:Med_ = ~0 h^-1^). Degradation increases in the LB pH 7.0 (β + β_pH_-β_Med_-β_pH:Med_ =0.029 h^-1^) and M9 pH 6.0 (β-β_pH_ + β_Med_-β_pH:Med_ =0.029 h^-1^) conditions, while in M9 pH 7.0 degradation further increases because of the individual contributions of pH, medium and their interaction (β + β_pH_ + β_Med_ + β_pH:Med_ =0.08 h^-1^). Taken together, the results indicate that pH and medium can tune HSL half life from ~9 hours (M9 pH 7.0) to a time that is much longer than the performed experiments, which are 32-hour long (LB pH 6.0).

**Figure 4:**
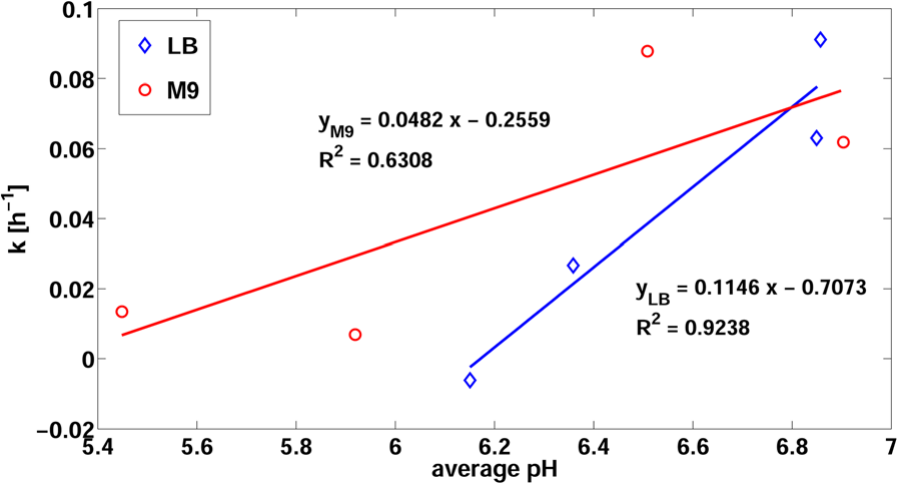
**HSL degradation rate (k) as a function of the average pH of bacterial culture in LB or M9.** Data points represent the estimated decay rates as a function of pH, taken from Table 1 and Additional file 1: Figure S10, respectively. Solid lines represent data fitting via regression line with the indicated equation and R^2^.

HSL decay data from cultured broth conditions were then considered. Figure 4 shows the HSL degradation rates (k) reported in Table 1, measured in the eight cultured broth conditions, as a function of the average pH of a culture in those conditions, computed from the data shown in Additional file 1: Figure S10.

As expected, in both the LB and M9 conditions there is a significant pH-dependent trend (P < 0.05, t-test). Moreover, k is systematically higher in M9 than in LB for a wide range of pH. These results confirm the significant contribution of pH and growth medium on HSL decay. However, since the linear regression model was fitted on sterile broth data, the precise prediction of HSL degradation in cultured broth may not be inferred by the model, although a similar medium- and pH-dependent trend is qualitatively observed.

## Discussion

The decay rates of the commonly used chemical inducers IPTG, ATc and HSL have been studied in different conditions. Temperature has been found to be the major factor affecting ATc, increasing degradation rate by 2-fold from 30°C to 37°C, while the other significant factors (medium and sterility) gave a minor contribution (<1.3-fold). In light of the reported results for ATc, the measured degradation rate is mainly dependent on non-biological processes as the decay occurs in sterile media, even if the presence of MG1655-Z1 culture slightly increases its degradation by 1.26-fold.

Medium and pH have been found to highly affect HSL in sterile broth, with a significant interaction: while no significant degradation occurs in LB pH 6.0 over a 32-h experiment, the increase of pH from 6.0 to 7.0 or the use of M9 instead of LB yield a significant degradation, which further increases in the M9 pH 7.0 condition. Data from cultured broth experiments qualitatively confirmed the medium and pH contribution. This study of HSL degradation confirms the important contribution of pH previously found in literature [22,23]. Temperature was previously identified as a key factor for the enzyme-independent hydrolysis of HSL, yielding a significantly higher degradation rate at 37°C than at 22°C [22], but in the temperature conditions used here (30°C and 37°C) no difference in degradation was detected.

Finally, no significant degradation was observed for IPTG among the tested conditions, thus confirming the high stability of this molecule, previously reported in high cell density cultures in different conditions [16,19,20].

The two growth media considered in this work, LB and M9, are widely used in biotechnological research and, in particular, in synthetic biology: plasmid propagations are usually performed in LB [28], while many quantitative expression studies are done in LB or M9 [1,2,4,7,38]. The latter is usually preferred when using fluorescent proteins as reporters because of its low background fluorescence. While the aim of our study was to provide useful data to support the predictable tuning of gene expression, our results cannot be directly exploited when different media are used, e.g., in case of high cell density cultures for microbial production of recombinant proteins where defined media are employed [19]. For this reason, additional measurements and linear model update should be performed when dealing with a condition of interest not included in this study.

Here, whole-cell biosensors have been an important tool for the quantification of the molecules of interest, with easy to carry out protocols that are compatible with multi-well microplates. However, it is worth noting that biosensors may provide inaccurate measurements when the culture environment of the samples unpredictably affects the biosensor output. To limit this effect, in this work we always prepared standard curves in the same growth medium of the samples and we also found no significant difference in biosensors activity when sterile media or exhausted media, with a known amount of inducer, were used as samples (see Additional file 1: Figure S3), thus demonstrating that measurements in the two extreme conditions of the same medium could be correctly performed. Such effects should always be tested when designing new experiments, as they could be dependent on strain, medium, growth conditions, inducer or biosensor. For all these reasons, the results reported in this work are valid under the assumption that only the inducer present in the sample determines the biosensors output, while other factors (e.g., degradation products) are assumed not to significantly affect it. To support the results, HPLC was used to confirm the biosensor measurements of ATc in a representative condition. The results obtained via HPLC were consistent with the measurements performed via the ATc biosensor. Other analytical methods, such as mass spectrometry, could be used to further confirm the obtained results.

The three inducers considered in this work are commonly known to be diffusible through bacterial cell membranes and for this reason intracellular and extracellular concentration of inducers are expected to be the same at equilibrium [19,22,39]. However, the “entrapment” of inducers in cells may affect inducer decay rate measurements in cultured broth conditions. This can occur if intracellular proteins which bind inducers are expressed, thus sequestering free inducer molecules. This effect may lead to overestimation of inducer decay rate and may be important when cells are at a high density, intracellular proteins are abundant or protein-inducer affinity is high. In the experiments carried out in this work in cultured broth conditions, HSL degradation assays are not affected by this phenomenon, as the MG1655-Z1 strain does not express LuxR, which specifically binds HSL. MG1655-Z1 over-expresses LacI, which may contribute, together with LacY, to IPTG entrapment in cells [20]. Since the MC1061 strain does not contain the lacI gene, also IPTG degradation assays in this strain are not affected. According to its genotype, the DB3.1 strain contains the wild-type lacI gene, but no significant concentration decrease is seen from the analysis of supernatants. Measurements may be affected by this phenomenon in ATc decay assays, since MG1655-Z1 over-expresses TetR, which binds ATc molecules. Importantly, inducer molecules could also bind other unspecific intracellular proteins or factors [20]. The quantification of bound inducer is not trivial and cannot be performed via the whole-cell biosensors, HPLC or mass spectrometry methods used or cited above [20]. Other works may be performed to elucidate the possible role of intracellular sequestration of inducers.

Finally, even though IPTG is not degraded and the pH-dependent HSL degradation has been well studied previously, the medium-dependent decay of HSL and ATc, the temperature-dependent decay of ATc, as well as the contribution of *E. coli* cultures on HSL and ATc degradation in cultured broth conditions are not known. Additional studies with different analytical methods could gain insight into the degradation mechanisms, while an explanation of the decay processes or the identification of the degradation products of the inducers of interest is beyond the scope of this work.

In summary, our study has provided a quantification for the inducer decay rates in different conditions, some of which were not tested in published works. Thanks to the factorial study design, a statistics-based framework has been used to find the main effects and their interactions affecting the decay rates. This led to the identification of previously unreported environmental factors and provided insights into the degradation mechanisms of the studied inducers. Despite the empirical nature of the reported results, the knowledge of degradation rate in several contexts can support the rational design of synthetic biological systems by improving the predictability of induction effects, especially for prolonged experiments.

## Competing interest

The authors declare that they have no competing interests.

## Authors’ contributions

NP, LP, SZ and PM conceived the study, designed the experiments and analyzed the data. NP performed the quantitative experiments. NP and SZ constructed the plasmids. SZ, MC, and GM designed and performed the measurements with HPLC. LP, MGCDA and PM wrote the paper. All authors read and approved the final manuscript.

## Supplementary Material

Additional file 1: Figure S1 Calibration curve of the IPTG (A), ATc (B) and HSL (C) biosensors in a representative experiment. **Figure S2.** Reproducibility of the IPTG (A), ATc (B) and HSL (C) biosensors measurements in different days. **Figure S3.** Effect of exhausted vs fresh medium on the activity of the IPTG (A), ATc (B) or HSL (C) biosensors. **Figure S4.** Data and fitting in IPTG degradation assays. **Figure S5.** Data and fitting in ATc degradation assays. **Figure S6.** Data and fitting in HSL degradation assays. **Figure S7.** Growth curves in cultured broth conditions. **Figure S8.** Quantification of the main factors and their interaction terms affecting the degradation of ATc in sterile broth. **Figure S9.** ATc measurements via HPLC: peaks of samples with 500 ng/ml, 350 ng/ml, 50 ng/ml and 10 ng/ml of ATc (A); standard calibration curve (B) and comparison between HPLC and biosensor measurements (C). **Figure S10.** pH of cultured broths. **Table S1.** Parameters of the linear model for ATc considering only the sterile broth conditions. Click here for file
