# Supplementary material for: Half-life measurements of chemical inducers for recombinant gene expression
Source: J Biol Eng. 2014 Feb 1;8:5. doi: 10.1186/1754-1611-8-5 (PMC3940292; doi:10.1186/1754-1611-8-5)
Supplement: Additional file 1: Figure S1 — Calibration curve of the IPTG (A), ATc (B) and HSL (C) biosensors in a representative experiment. Figure S2. Reproducibility of the IPTG (A), ATc (B) and HSL (C) biosensors measurements in different days. Figure S3. Effect of exhausted vs fresh medium on the activity of the IPTG (A), ATc (B) or HSL (C) biosensors. Figure S4. Data and fitting in IPTG degradation assays. Figure S5. Data and fitting in ATc degradation assays. Figure S6. Data and fitting in HSL degradation assays. Figure S7. Growth curves in cultured broth conditions. Figure S8. Quantification of the main factors and their interaction terms affecting the degradation of ATc in sterile broth. Figure S9. ATc measurements via HPLC: peaks of samples with 500 ng/ml, 350 ng/ml, 50 ng/ml and 10 ng/ml of ATc (A); standard calibration curve (B) and comparison between HPLC and biosensor measurements (C). Figure S10. pH of cultured broths. Table S1. Parameters of the linear model for ATc considering only the sterile broth conditions. [file 1754-1611-8-5-S1.pdf]

SUPPLEMENTARY INFORMATION

Supplementary figures:

Figure S1 - Calibration curve of the IPTG (A), ATc (B) and HSL (C) biosensors in a representative experiment. Each curve was obtained in a single representative experiment (performed as described in the Methods section). In all the three experiments, serial dilutions of the calibration samples were prepared in M9 at pH 7.0. The final inducer concentrations in the microplate well are reported in the X-axis, while the corresponding S_{cell} values (two replicates) are reported in the Y-axis (circles). Solid line represents data fitting with a Hill function, as described in the Methods section.

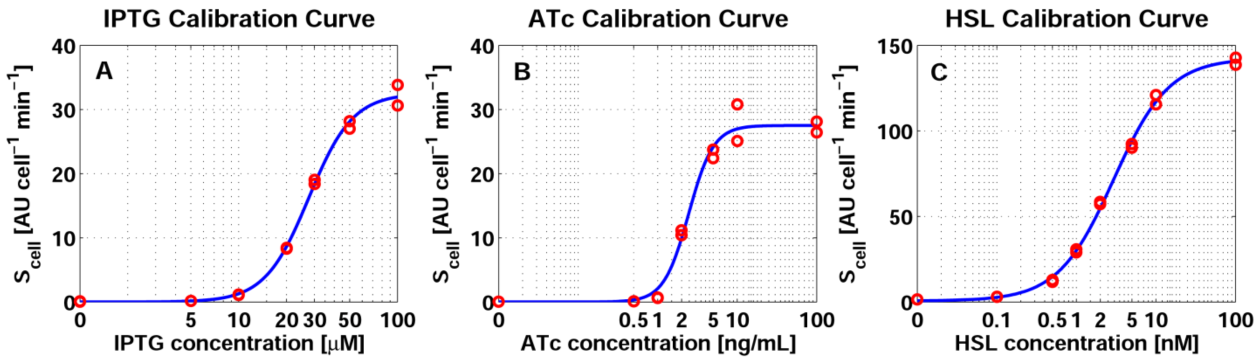

Figure S2 - Reproducibility of the IPTG (A), ATc (B) and HSL (C) biosensors measurements in different days. For each biosensor, the inducer concentration of a set of samples was measured in two different days. Measured concentration of day 1 and day 2 is reported in the X- and Y- axis, respectively. Circles represent data points, while solid line represents the Y=X line.

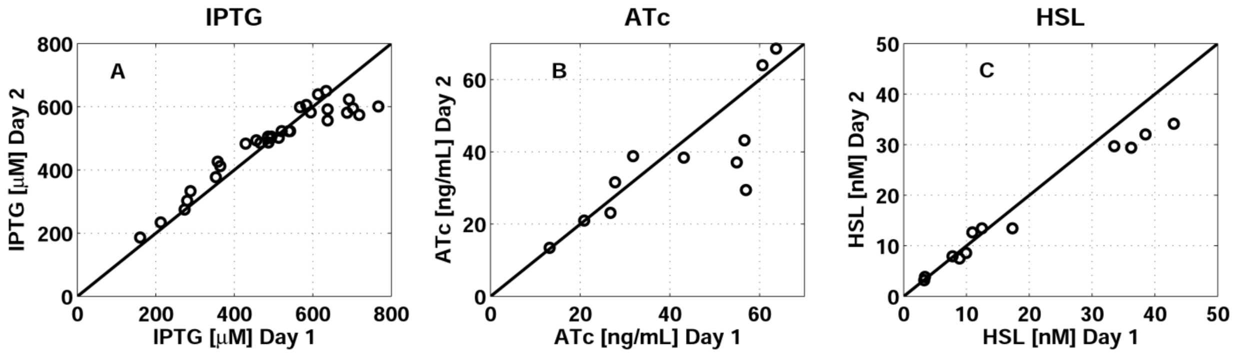

Figure S3 - Effect of exhausted vs fresh medium on the activity of the IPTG (A), ATc (B) or HSL (C) biosensors. The activity of the three biosensors was measured in terms of S_{cell} when assaying samples of known concentrations of IPTG, ATc or HSL. Inducers were diluted in different fresh (LB pH 6.0, LB pH 7.0, M9 pH 6.0 and M9 pH 7.0) or exhausted (from the supernatant of 32-h cultures grown at 37°C in LB pH 6.0, LB pH 7.0, M9 pH 6.0 and M9 pH 7.0) media. Assays were performed as described in the Methods section, except that the cultures used to prepare the exhausted media were grown in absence of inducers. The final concentration of IPTG, ATc and HSL in the microplate wells was 20 µM, 2 ng/ml and 5 nM, respectively. Bars represent the average of at least two independent replicates, while error bars represent the 95% confidence intervals of the average value.

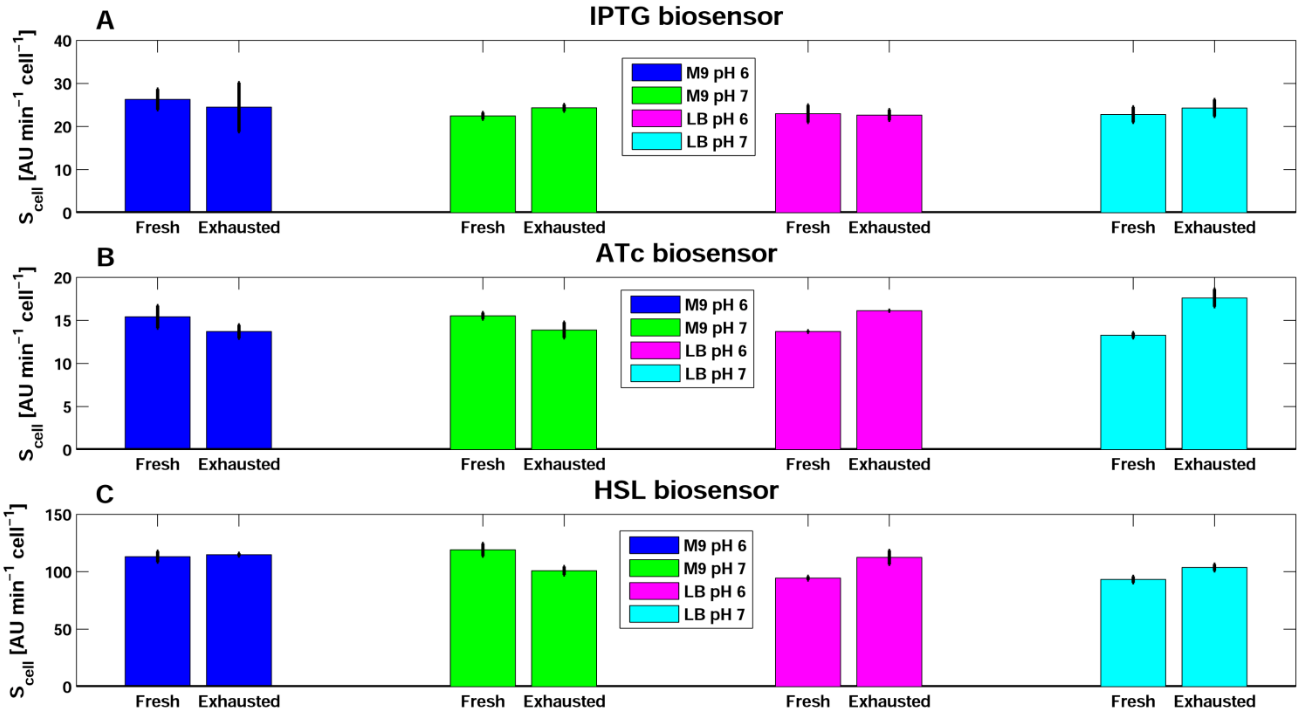

Figure S4 - Data and fitting in IPTG degradation assays. Fitting of IPTG measurements over time during the performed degradation assays. Data points represent the average concentration values, error bars represent the 95% confidence intervals of the average value of at least two independent experiments and solid lines are the fitted first-order exponential model curves.

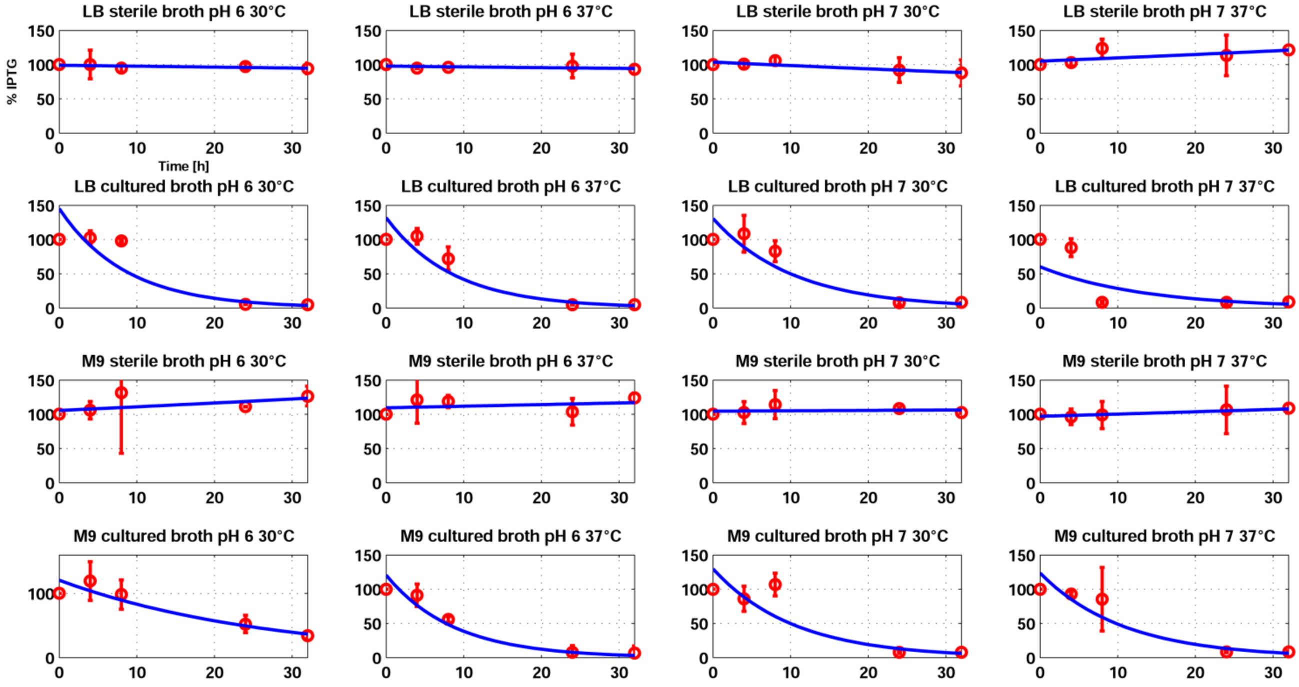

Figure S5 - Data and fitting in ATc degradation assays. Fitting of ATc measurements over time during the performed degradation assays. Data points represent the average concentration values, error bars represent the 95% confidence intervals of the average value of at least two independent experiments and solid lines are the fitted first-order exponential model curves.

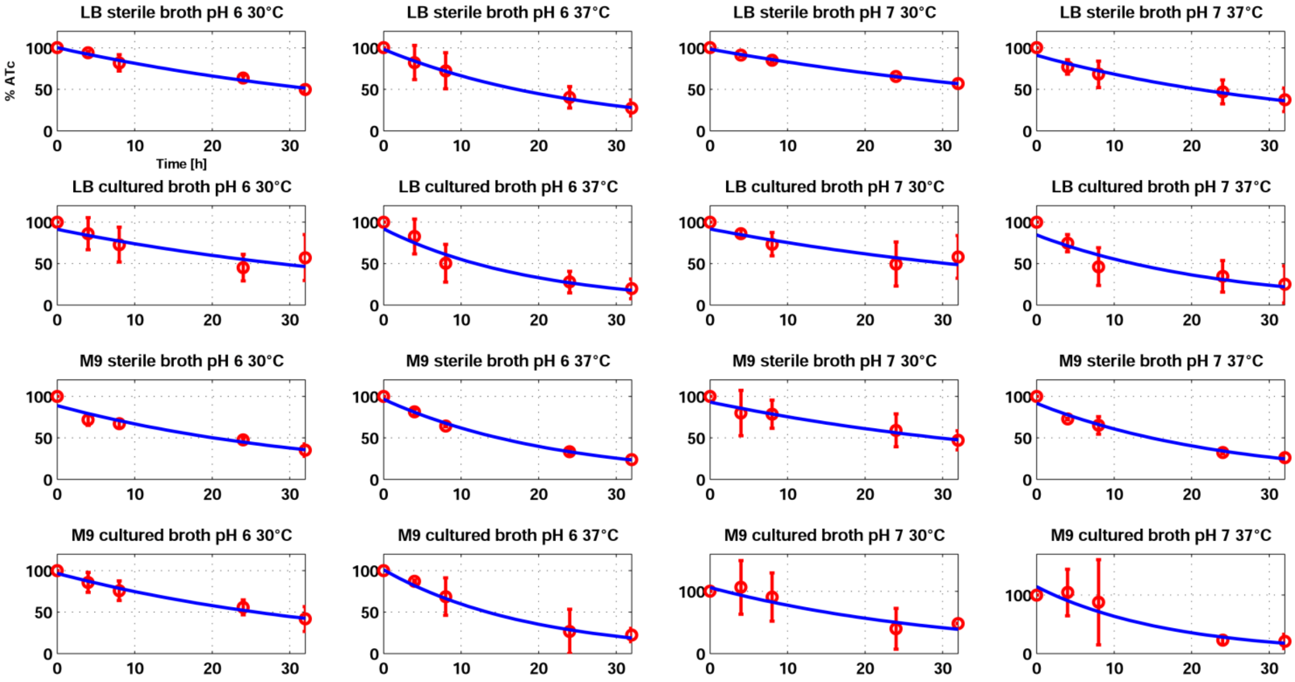

Figure S6 - Data and fitting in HSL degradation assays. Fitting of HSL measurements over time during the performed degradation assays. Data points represent the average concentration values, error bars represent the 95% confidence intervals of the average value of at least two independent experiments and solid lines are the fitted first-order exponential model curves.

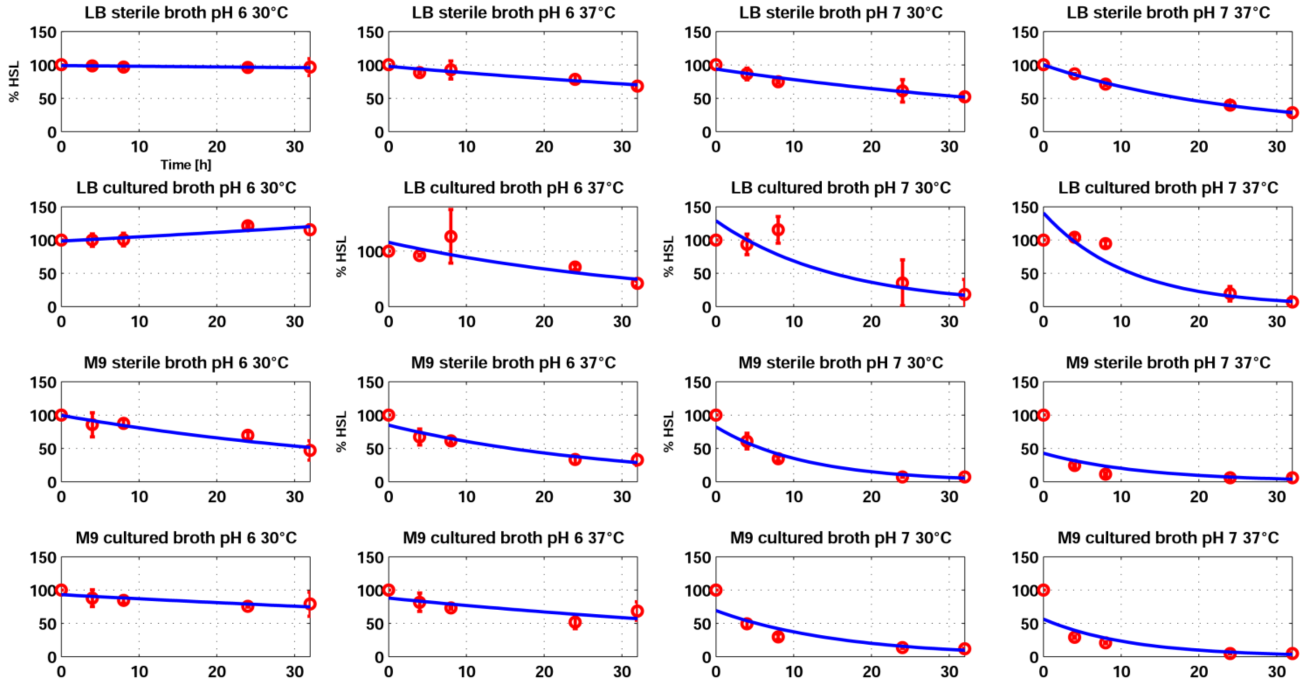

Figure S7 - Growth curves in cultured broth conditions. Growth curves are shown for a representative experiment in a set of medium, temperature and pH conditions. The shown measurements, expressed as optical density at 600 nm over time, come from an assay for IPTG. Data points represent the OD600 values for the reported conditions.

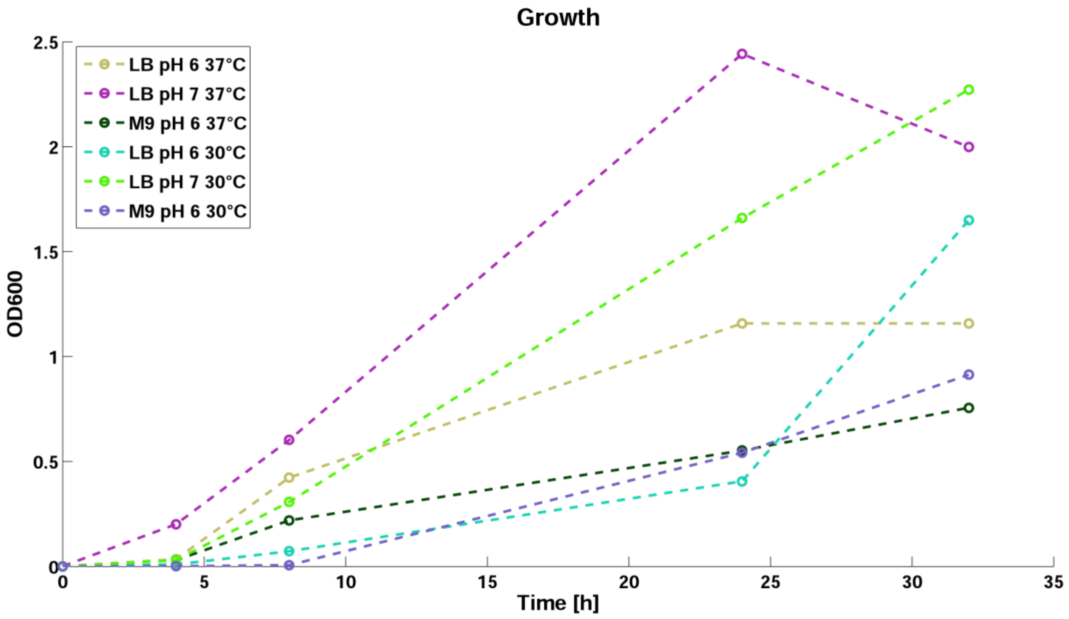

Figure S8 - Quantification of the main factors and their interaction terms affecting the degradation of ATc in sterile broth. The pie chart shows the variability explained by each of the considered factors and interaction terms (SS_j, Eq.4). 100% of the pie corresponds to the residual error of the null model (SS_{tot}, Eq.3), while the error term represents the residual error that remains unexplained by the full linear model (SSE, Eq.5). Colon indicates an interaction between two factors.

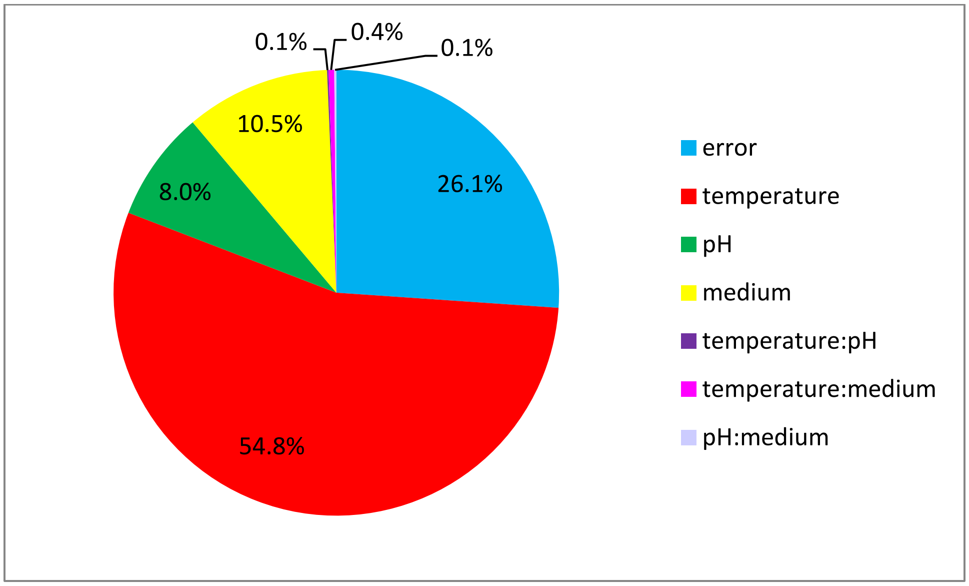

Figure S9 - ATc measurements via HPLC: peaks of samples with 500 ng/ml, 350 ng/ml, 50 ng/ml and 10 ng/ml of ATc (A); standard calibration curve (B) and comparison between HPLC and biosensor measurements (C). Panel A shows the specific peak of ATc, starting at the retention time of 14.26 min, for samples with different inducer concentrations. No detectable peak is observed in samples without ATc (data not shown). Panel B shows the standard calibration curve, expressing ATc concentration vs ATc peak area, and the corresponding regression line (R^2>0.99). Red circles are the average values and blue squares indicate the 95% confidence intervals of the average value of three independent measurements. The linearity range lower limit of the curve is 50 ng/ml (data not shown). Samples of panels A and B were prepared by adding known amounts of ATc to sterile M9 at pH 7.0. Panel C shows the result of an ATc decay experiment in M9 cultured broth at 30°C, pH 7.0, when measured via HPLC (red) or ATc biosensor (blue). Circles represent the average values and error bars are the 95% confidence intervals of the average values of two independent experiments. Lines represent the fitted first-order exponential model curves. Data and fitted curves are shown in absolute units (ng/ml of ATc). Results show good agreement between HPLC and biosensor measurements (CV of 8%) and fitted decay rates (k=0.013±0.004 for HPLC and k=0.011±0.006 for biosensor).

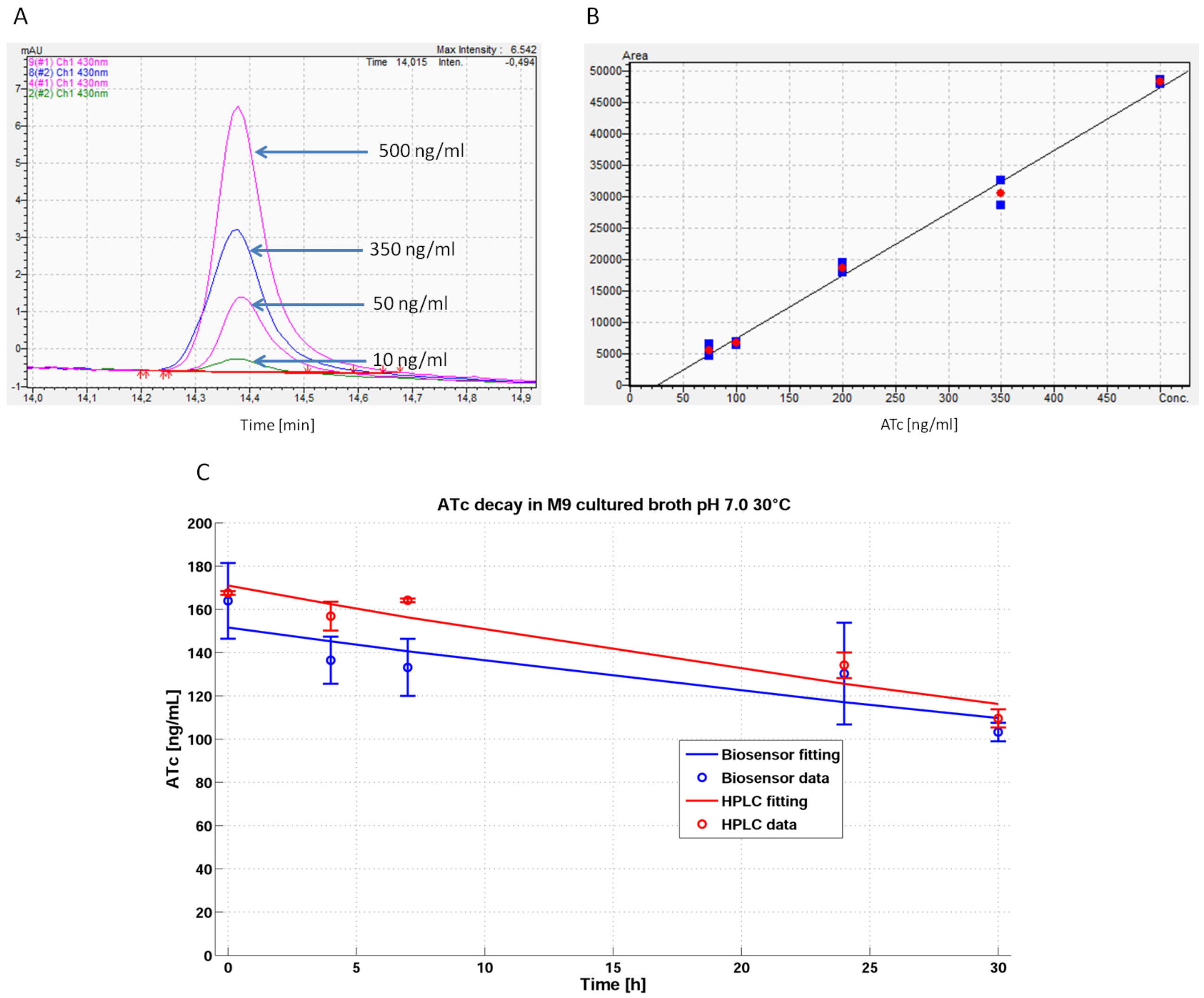

Figure S10 - pH of cultured broths. Because the presence of a growing bacterial population can affect the pH of the medium, pH measurements were taken at specific time points in cultured broth experiments. Data points represent the measured pH value of a representative experiment or the average value of 8-10 replicates. In the latter case, error bars represent their 95% confidence intervals. Panel A: growth at 30°C; panel B: growth at 37°C.

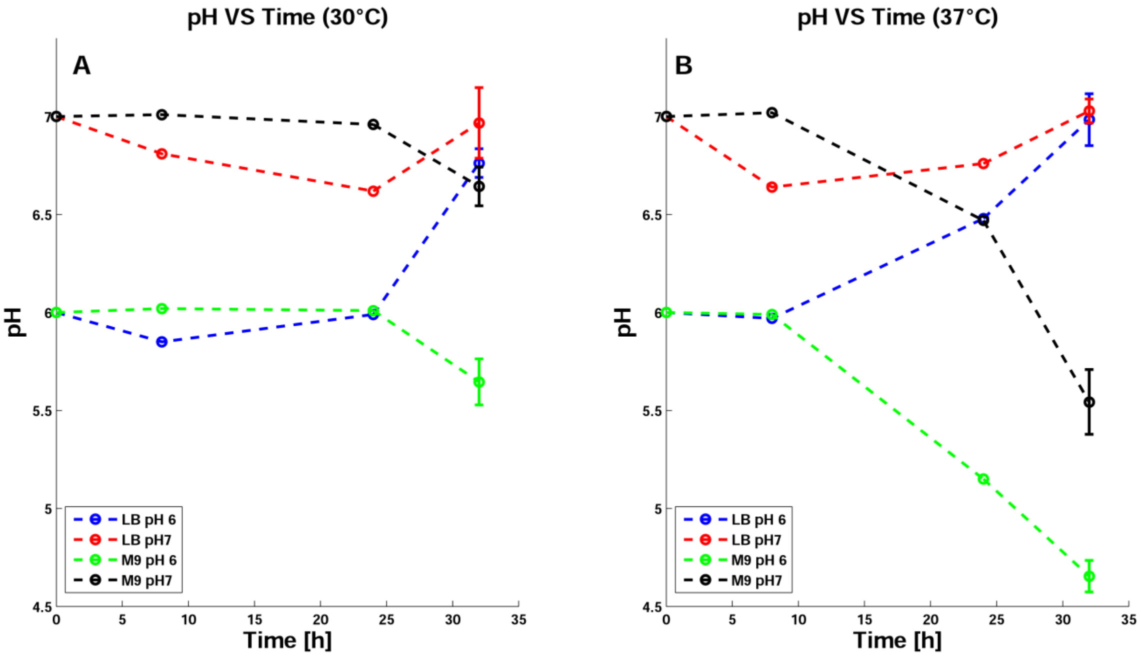

Supplementary tables:

Table S1 - Parameters of the linear model for ATc considering only the sterile broth conditions. Estimated regression coefficients and p-values are reported for the study of ATc in sterile broth. The regression coefficients β (i.e., the coefficients that are multiplied by time) shown in bold are significantly different from zero (P<0.05, t-test) and identify the main factor or interaction terms that significantly contribute to inducer decay.

Parameter	Identified value	p-value
α	4.551	1.48e-98
α_{Temp}	-0.004049	0.83
α_{pH}	-0.01258	0.51
α_{Med}	-0.02341	0.23
$\alpha_{Temp \cdot pH}$	-0.01977	0.31
$\alpha_{Temp \cdot Med}$	0.02053	0.29
$\alpha_{pH \cdot Med}$	0.01120	0.56
$\boldsymbol{\beta_t}$	**0.03016**	**3.5e-39**
$\boldsymbol{\beta_{Temp}}$	**0.008235**	**4.1e-11**
$\boldsymbol{\beta_{pH}}$	**-0.003148**	**3.6e-3**
$\boldsymbol{\beta_{Med}}$	**0.003604**	**9.7e-4**
$\beta_{Temp \cdot pH}$	-0.0003665	0.73
$\beta_{Temp \cdot Med}$	0.0007120	0.50
$\beta_{pH \cdot Med}$	0.0004307	0.68
